# Continuous Renal Replacement Therapy and Extracorporeal Membrane Oxygenation in Patients with Cardiogenic Shock: Results from the Rescue Registry

**DOI:** 10.3390/jcm14051498

**Published:** 2025-02-24

**Authors:** Chewan Lim, Young Hak Chung, Chul-Min Ahn, Sungsoo Cho, Jeong Hoon Yang, Tae Soo Kang, Sang-Hyup Lee, Yong-Joon Lee, Seung-Jun Lee, Sung-Jin Hong, Jung-Sun Kim, Byeong-Keuk Kim, Young-Guk Ko, Donghoon Choi, Hyeon-Cheol Gwon, Myeong-Ki Hong, Yangsoo Jang

**Affiliations:** 1 Division of Cardiology, Department of Internal Medicine, International St. Mary’s Hospital, Catholic Kwandong University College of Medicine, Incheon 22711, Republic of Korea; chewan0329@gmail.com; 2 Division of Cardiovascular Medicine, Department of Internal Medicine, Dankook University Hospital, Dankook University College of Medicine, Cheonan 31116, Republic of Korea; younghak2016@naver.com (Y.H.C.);; 3 Division of Cardiology, Department of Internal Medicine, Severance Cardiovascular Hospital, Yonsei University College of Medicine, Seoul 03722, Republic of Korea; shlee0917@yuhs.ac (S.-H.L.);; 4 Division of Cardiology, Department of Internal Medicine, Gangnam Severance Hospital, Yonsei University College of Medicine, Seoul 03722, Republic of Korea; 5 Division of Cardiology, Department of Internal Medicine, Heart Vascular Stroke Institute, Samsung Medical Center, Sungkyunkwan University School of Medicine, Seoul 06351, Republic of Korea; 6 Department of Cardiology, CHA Bundang Medical Center, CHA University, Seongnam 13496, Republic of Korea

**Keywords:** cardiogenic shock, continuous renal replacement therapy, extracorporeal membrane oxygenation, acute kidney injury, mortality

## Abstract

**Background:** Cardiogenic shock (CS) frequently leads to multiorgan failure, often necessitating continuous renal replacement therapy (CRRT) or extracorporeal membrane oxygenation (ECMO). We evaluated the association between CRRT, ECMO, and its prognostic implication in patients with CS. **Methods:** A total of 1247 patients with CS were enrolled from the RESCUE (Retrospective and Prospective Observational Study to Investigate Clinical Outcomes and Efficacy of Left Ventricular Assist Device for Korean Patients with Cardiogenic Shock) registry between January 2014 and December 2018. The primary outcomes, including the 72 h and 30-day all-cause mortality rates, were analyzed in relation to the use of ECMO and CRRT among CS patients. **Results:** Among 751 non-ECMO patients, 90 (12%) underwent CRRT, while among 496 ECMO patients, 195 (39.3%) underwent CRRT. Overall, CRRT was associated with higher 30-day mortality. However, among ECMO patients, CRRT was linked to lower 72 h mortality (19.6% versus 12.3%; *p* = 0.045). Multivariate analysis showed that CRRT reduced 72 h mortality in ECMO patients (hazard ratio: 0.44; 95% confidence interval: 0.21–0.91; *p* = 0.027). Independent predictors for CRRT included an estimated GFR < 44 mL/min/1.73 m^2^, mechanical ventilation, ECMO use, IABP use, and increased lactate. **Conclusions:** CS patients receiving CRRT had higher 30-day mortality. Nonetheless, CRRT administration was more common in ECMO patients, potentially improving early in-hospital clinical outcomes.

## 1. Introduction

Cardiogenic shock (CS) is a life-threatening condition often accompanied by noncardiac organ failure [1]. The kidney is one of the most susceptible organs to ischemia, with approximately 30%–50% of patients with CS experiencing renal damage, often manifesting as acute kidney injury (AKI) [2]. Renal function is particularly sensitive to sudden reductions in cardiac output (CO), where acute renal hypoperfusion promptly diminishes glomerular filtration rate, urine output, and parenchymal oxygenation, frequently precipitating AKI [3]. In this context, renal hypoperfusion may be exacerbated by venous congestion, amplifying backward pressure and further diminishing the glomerular filtration rate [4]. Moreover, in 10%–25% of patients with CS who do not respond to conventional diuretic therapy, renal replacement therapy may be necessary to preserve kidney function, particularly in severely compromised renal conditions [5]. In cases of refractory CS, patients often require extracorporeal membrane oxygenation (ECMO), but this is associated with risks such as fluid overload, ischemic/reperfusion injury, systemic inflammation, hemolysis, and the worsening of AKI [6].

Continuous renal replacement therapy (CRRT) serves to address fluid overload, uremia, acid–base abnormalities, and electrolyte imbalance in critically ill patients, particularly those with CS. The incorporation of ECMO or CRRT is frequently indispensable for managing and providing comprehensive support for critically ill patients with CS [7,8]. In CS patients necessitating ECMO, concurrent AKI often necessitates CRRT intervention [9]. Nevertheless, there is limited research examining the overall association and prognostic implications of these two mechanical support modalities in CS patients.

Therefore, this study aimed to evaluate the impact of CRRT and ECMO in CS patients using a multicenter, dedicated CS registry.

## 2. Materials and Methods

### 2.1. Study Population

The study population was enrolled from the RESCUE (Retrospective and Prospective Observational Study to Investigate Clinical Outcomes and Efficacy of Left Ventricular Assist Device for Korean Patients with Cardiogenic Shock) (NCT02985008 at https://www.clinicaltrials.gov/ (accessed on 5 December 2016.)) registry between January 2014 and December 2018 [10].

The inclusion criteria were as follows: patients who (1) had a systolic blood pressure < 90 mmHg for 30 min despite adequate fluid resuscitation or who required inotropes or vasopressors to maintain a systolic blood pressure >90 mmHg or (2) had signs of impaired peripheral organ perfusion, with any of the following: altered mental status, cold extremities, a urine output <0.5 mL/kg/h, a serum lactate level ≥ 2.0 mmol/L, or acute pulmonary edema. The exclusion criteria included patients who experienced shock that was not of primary cardiac origin, who experienced shock accompanied by out-of-hospital cardiac arrest, who were allergic to heparin, or who refused active medical management. Finally, 1247 patients (>19 years) with CS were included in the analysis.

The institutional review board of each hospital approved the study protocol and waived the requirement for written informed consent for patients enrolled in the retrospective registry (n = 954). In the prospective enrollment arm (n = 293), all patients or their legally authorized representatives provided written informed consent, and all information was collected prospectively. The study was performed in accordance with the Good Clinical Practice Guidelines and the principles of the Declaration of Helsinki.

### 2.2. CRRT and ECMO Management

The decision to start CRRT was based on the presence of ≥1 criterion used in critically ill patients [11,12], including AKI with prolonged (>24 h) oligoanuria, overt heart failure (acute pulmonary edema), an increase in azotemia (≥200 mg/dL), severe hyperkalemia (>6.5 mEq/L or lower when associated with typical electrocardiographic abnormalities), and metabolic acidosis (pH < 7.1). CRRT is usually performed using blood flow rates of 100–150 mL/min through a double-lumen 12F catheter inserted into the femoral or jugular vein. The final decision to initiate CRRT, as well as the choice of CRRT modality, was made after consultation with a nephrologist.

Mechanical hemodynamic support using ECMO was considered based on current guideline recommendations [13]. Management during ECMO support followed the Extracorporeal Life Support Organization guidelines [14]. Peripheral venoarterial (VA)-ECMO cannulation was the most frequently used access method, performed via the common femoral artery and vein, just below the inguinal ligament and above their respective bifurcations. A 15F–17F arterial cannula and a 21F–28F venous cannula were used to supply sufficient flow based on the patient’s needs. Continuous intravenous unfractionated heparin was administered to maintain an activated clotting time between 150 and 180 s or between 180 and 220 s, depending on each hospital’s protocol, provided there was no contraindication. When inserting a distal perfusion catheter, a fluoroscopy- or ultrasound-guided approach was used. VA-ECMO weaning was considered when patients were hemodynamically stable without any vasopressors or with a low level of pharmacological support (norepinephrine, ≤0.05 mg/kg per minute; and/or dobutamine, ≤5 mg/kg per minute) and had a mean arterial pressure ≥ 65 mmHg, lactate < 2 mmol/L, and central vein pressure ≤ 15 mmHg.

### 2.3. Definitions and Outcomes

The primary endpoints included all-cause mortality at 72 h and 30 days following the onset of CS. The secondary endpoints included 1-year all-cause mortality, readmission for heart failure (HF), revascularization, and incidence of cerebrovascular accidents (CVA). Clinical outcomes were documented through clinic visits or telephone interviews with patients at 1 and 12 months. The durations of intensive care unit (ICU) and hospital stays up to discharge were evaluated for all survivors.

### 2.4. Statistical Analysis

Continuous variables are expressed as the mean ± standard deviation or median (interquartile range) and were compared using Student’s *t* test. Categorical variables are expressed as frequencies (percentages) and were compared using the chi-square test or Fisher’s exact test. Univariate and multivariate Cox proportional hazards regression analyses were performed to identify independent predictors and calculate the 72 h and 30-day mortality risk.

In the multivariable models, the covariates included were age, sex, hypertension, diabetes status, previous myocardial infarction (MI), peripheral arterial occlusive disease (PAOD), previous cerebrovascular accident (CVA), lactate, estimated glomerular filtration rate (GFR), intra-aortic balloon pump (IABP), vasoactive inotropic score, extracorporeal cardiopulmonary resuscitation (ECPR), and mechanical ventilation. In many cases, CRRT was initiated based on clinical judgment without follow-up laboratory tests. To assess baseline renal function, we categorized the estimated glomerular filtration rate (eGFR) into the following stages: ≤30 mL/min/1.73 m^2^, >30 to ≤45 mL/min/1.73 m^2^, >45 to ≤69 mL/min/1.73 m^2^, and ≥70 mL/min/1.73 m^2^.

To minimize the impact of potential confounding variables, we applied the inverse probability of treatment weighting (IPTW) method. This approach was selected over propensity score (PS) matching due to the significant imbalance in the number of patients between the ECMO and non-ECMO groups. IPTW, which is essentially the inverse of the PS, assigns weights to patients in inverse proportion to their probability of receiving the treatment. As a result, patients who are more likely to receive treatment are weighted lower, while those less likely to receive treatment are weighted higher. The covariates used in the IPTW analysis were the same as those included in the multivariable analysis. To assess the balance of covariates after IPTW, we presented the baseline characteristics and standardized mean differences for all covariates (Supplementary Table S3). Finally, we constructed IPTW-adjusted Cox proportional hazard regression models to estimate the adjusted hazard ratios (HRs) for the variable of interest in relation to the risk of death.

Cumulative incidences of clinical events are presented as a Kaplan–Meier curve based on the time of performing the index procedure to the occurrence of the first event of interest during follow-up. We further performed landmark analyses with 72 h and 30-day all-cause mortality in CS patients without ECMO and with ECMO.

Statistical analyses were performed using SPSS version 25 for Windows (SPSS-PC, Chicago, IL, USA) and R version 3.6.2 (R Foundation for Statistical Computing, Vienna, Austria). All tests were two-tailed, and *p* < 0.05 was considered to indicate statistical significance.

## 3. Results

### 3.1. Baseline Characteristics

**Figure 1:**
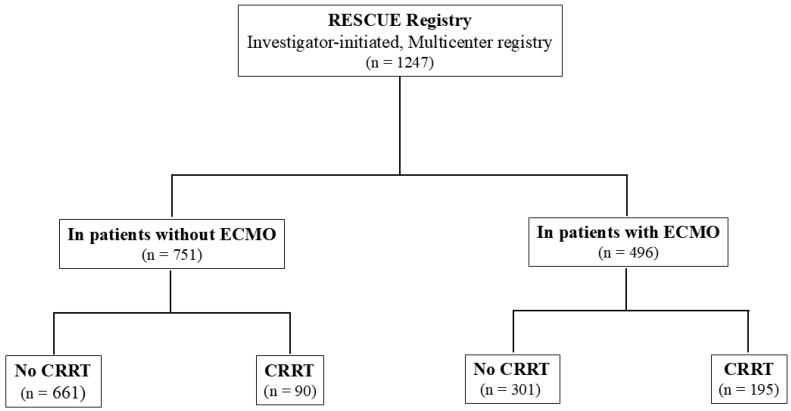
Study Flow. CRRT, continuous renal replacement therapy; ECMO, extracorporeal membrane oxygenation.

Patients were categorized into four groups based on whether they received ECMO or CRRT. Among the 751 CS patients who did not receive ECMO, 90 (12%) patients underwent CRRT, while among the 496 CS patients who received ECMO, 195 (39.3%) patients underwent CRRT (Figure 1).

Ischemic cardiomyopathy (ICMP) was a major cause of CS regardless of receiving ECMO or CRRT. Myocarditis was more frequently the cause of CS in patients receiving ECMO than in those not receiving ECMO (0.7% vs.7.1%, respectively) (Table 1).

Among patients who did not receive ECMO, those in the CRRT group were older and more likely to be female, have hypertension, have diabetes, have peripheral artery disease, have chronic kidney disease (CKD), be smokers, and be on mechanical ventilation than those in the non-CRRT group. Within the CRRT group, significantly lower levels of hemoglobin, platelet count, alanine aminotransferase, serum sodium, and estimated GFR were observed, while creatinine, lactate, NT-proBNP, and vasoactive scores were significantly greater compared to the non-CRRT group. Among patients receiving ECMO, those in the CRRT group had a greater incidence of CKD, previous coronary artery bypass graft (CABG), elevated baseline levels of creatinine and lactic acid, and reduced platelet count and eGFR. Additionally, a greater proportion of patients in the CRRT group than in the non-CRRT group underwent mechanical ventilation (Table 1 and Supplementary Materials, Table S1).

### 3.2. Clinical Outcomes

In the overall population, a prolonged ICU stay and prolonged hospitalization duration were observed in the CRRT group. There was no significant difference in 72 h all-cause mortality between the non-CRRT and CRRT groups. Thirty-day all-cause mortality was significantly greater in the CRRT group than in the non-CRRT group (Supplementary Materials, Table S2 and Figure S1).

**Figure 2:**
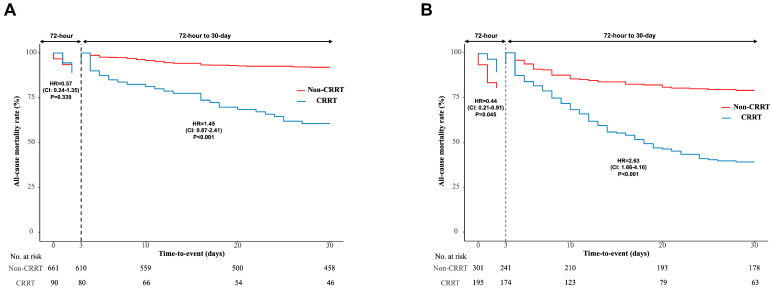
Landmark analysis of 72 h and 30-day all-cause mortality in CS patients without ECMO (**A**) and with ECMO (**B**). CI, confidence interval; CRRT, continuous renal replacement therapy; ECMO, extracorporeal membrane oxygenation; HR, hazard ratio.

Among patients who did not receive ECMO, a prolonged ICU stay and prolonged hospitalization duration were evident in the CRRT group. Similarly, among patients who received ECMO, a longer ICU stay was observed in the CRRT group, although no difference in hospitalization duration was noted between the non-CRRT and CRRT cohorts. In patients who did not receive ECMO, there was no significant difference in 72 h all-cause mortality between the non-CRRT and CRRT groups (Table 2 and Figure 2A). However, among patients who received ECMO, 72 h all-cause mortality was notably lower in the CRRT group than in the non-CRRT group (59 (19.6%) vs. 24 (12.3%), *p* = 0.045) (Table 2 and Figure 2B). Furthermore, 30-day all-cause mortality and 1-year all-cause mortality rates were significantly greater in the CRRT group than in the non-CRRT group, regardless of ECMO utilization. There were no significant differences in the rates of admission for 1-year heart failure (HF), revascularization, or stroke between the two groups, regardless of ECMO utilization.

### 3.3. Multivariate and IPTW Analysis of Predictors of Clinical Outcomes

Table 3 and Table 4 present the results of multivariate Cox proportional survival analysis, identifying independent predictors for 72 h and 1-month all-cause mortality. Lactate level and the vasoactive-inotrope score emerged as consistent independent predictors for both 72 h and 30-day all-cause mortality, regardless of ECMO or CRRT utilization. Notably, CRRT was identified as an independent predictor associated with reduced 72 h all-cause mortality among patients undergoing ECMO (adjusted HR: 0.44; 95% confidence interval [CI]: 0.21–0.9; *p* = 0.027). However, CRRT was independently associated with increased 30-day all-cause mortality among patients undergoing ECMO (adjusted HR: 2.63; 95% CI: 1.66–4.16; *p* < 0.001).

In the IPTW analysis, the hazard ratios for 72 h and 1-month all-cause mortality for each covariate were consistent with the results of the multivariate Cox analysis. Table 5 presents a comparison of the impact of CRRT on mortality according to ECMO status, as assessed by multivariate Cox regression and IPTW analysis.

In the IPTW analysis, CRRT remained an independent predictor associated with a reduced risk of 72 h all-cause mortality among patients receiving ECMO (adjusted HR: 0.40; 95% CI: 0.19–0.86; *p* = 0.019). However, CRRT was independently associated with an increased risk of 30-day all-cause mortality in this patient group (adjusted HR: 1.77; 95% CI: 1.17–2.67; *p* = 0.006).

### 3.4. Independent Predictors of the Need for CRRT in CS Patients

Table 6 presents the independent predictors associated with the need for CRRT in CS patients. Factors such as increased lactate (odds ratio [OR]: 1.06, CI: 1.01–1.1), an estimated GFR < 44 mL/min/1.73 m^2^ (OR: 9.39, CI: 5.02–17.57), intra-aortic balloon pump (IABP) usage (OR: 1.46, CI: 0.91–2.32), the use of ECMO (OR: 3.59, CI: 2.4–5.38) and the need for mechanical ventilation (OR: 4.16, CI: 2.51–6.91) were identified as independent predictors of the need for CRRT in CS patients.

## 4. Discussion

The key observations from our analysis are summarized as follows: (1) Among patients with CS, a greater proportion required CRRT than those who received ECMO, in contrast to patients who did not receive ECMO. (2) Patients with CS who underwent CRRT exhibited elevated 30-day mortality rates compared to those who did not undergo CRRT. (3) CRRT was an independent predictor of reduced 72 h all-cause mortality in patients who underwent ECMO. (4) Independent predictors for the requirement of CRRT in CS patients included factors such as estimated GFR < 44 mL/min/1.73 m^2^, the necessity for mechanical ventilation, ECMO utilization, IABP utilization, and increased lactate.

In the context of CS, compromised ventricular function can precipitate AKI, characterized by diminished renal perfusion and heightened renal venous pressure, thereby highlighting the deterioration of renal function as a pivotal prognostic marker for short-term mortality [15]. While ECMO serves as a life-saving intervention for CS patients, an association has been documented between fluid overload during ECMO support and an increased risk of mortality [9]. Furthermore, recent meta-analyses have reported that exposure to severe hyperoxemia following the initiation of VA-ECMO may be associated with an approximately twofold increase in the likelihood of poor neurological outcomes and mortality [16]. The integration of CRRT with ECMO is paramount for effectively managing AKI and fluid overload [7]. However, previous studies have not conducted a comprehensive analysis to ascertain whether ECMO or CRRT was employed in patients with CS.

To our knowledge, this study represents the first comprehensive analysis of the interplay between ECMO and CRRT in patients with CS. In our study, among 751 CS patients who did not receive ECMO, 90 (12%) underwent CRRT, whereas among 496 CS patients who received ECMO, 195 (39.3%) underwent CRRT. Compared with those not receiving CRRT, CS patients receiving ECMO exhibited lower eGFRs and elevated lactate, as well as greater mechanical ventilation, suggesting more severe progression of AKI and resultant fluid overload in the CRRT group. Considering that ECMO can exacerbate fluid overload, the necessity for CRRT becomes apparent. Patients with CS exhibited a notably poor prognosis, characterized by an overall in-hospital mortality rate of 40%. Specifically, when ECMO was utilized, the 72 h mortality rate was 26%, with an in-hospital mortality rate of 60% [17,18]. Smith M et al. reported that survival on VA-ECMO is significantly influenced by support duration, with a notable increase in mortality observed within four days, possibly indicating early treatment failure [19]. Although the highest survival rates are observed when weaning from ECMO occurs on the fourth day, it is suggested that survival may decline beyond this point, reaching a relatively constant rate after the second week of ECMO support [19]. Hence, to mitigate multiorgan failure progression and manage fluid overload during the initial ECMO phase, CRRT may be indispensable, even in cases of maintained urine output. Our study focused on 72 h mortality as an early in-hospital clinical outcome measure. Notably, among CS patients undergoing ECMO, those in the CRRT group exhibited significantly lower 72 h all-cause mortality than those in the non-CRRT group. CRRT emerged as an independent predictor associated with reduced 72 h all-cause mortality in ECMO recipients. Consequently, the implementation of CRRT may hold promise for enhancing early in-hospital clinical outcomes.

Nevertheless, in our study, 30-day mortality was greater in the CRRT group than in the non-CRRT group, with CRRT identified as an independent contributor to increased 30-day all-cause mortality among patients undergoing ECMO. Consistent with prior studies, patients necessitating both ECMO and CRRT exhibited a notably elevated mortality rate. The independent risk factor for mortality in critically ill patients undergoing ECMO is the presence of AKI, with studies indicating a substantial increase in 1- to 3-month mortality in those requiring CRRT during ECMO treatment compared to those without CRRT [20].

Nonetheless, the independent association between positive fluid balance and mortality underscores the importance of prevention, with CRRT being deemed valuable for timely and precise control of patients’ fluid balances, particularly when an early approach is implemented. In our study, high lactate levels, low eGFRs, and mechanical ventilation were associated with an increased risk of CRRT implementation in patients with CS undergoing ECMO. It appears essential to identify these features in CS patients to appropriately apply CRRT.

In patients with CS undergoing ECMO, initial outcomes may improve following CRRT, but subsequent outcomes may deteriorate. This phenomenon may be linked to a poorer cardiac profile, a greater burden of comorbidities, and a longer ischemic duration in patients with CS requiring CRRT than in those without CS requiring CRRT [21,22]. Previous studies attributed the development of AKI in CS to heart–kidney crosstalk, known as cardiorenal syndrome (CRS), which comprises five subtypes [4,23]. Notably, type 1 CRS elucidates the pathophysiology of AKI development in acute worsening cardiac function scenarios such as CS. Conversely, acute deterioration of kidney function could contribute to acute cardiac dysfunction, as observed in type 3 CRS patients. Volume overload and increased blood pressure following AKI can burden cardiac muscle and aggravate the ischemic process. Furthermore, electrolyte imbalance and metabolic acidosis could cause a negative inotropic effect and arrhythmic events, in addition to the hemodynamic instability of CS. Inflammation and apoptosis may also damage cardiac muscle. These two CRS types (types 1 and 3) may coexist and aggravate each other, forming a vicious cycle and resulting in poor clinical outcomes [24,25]. Hence, in patients experiencing cardiogenic shock and undergoing mechanical circulatory support, prompt correction of abnormal hemodynamics and maintenance of adequate tissue perfusion are imperative to prevent organ dysfunction. In cases where these measures fail to restore optimal function and perfusion, timely consideration of interventions such as heart transplantation is crucial to ensure patient survival.

Our study had several limitations. First, this was a multicenter observational study with a retrospective-prospective design. Therefore, selection and observational biases were unavoidable. Second, although multivariate statistical methods, risk adjustment for survival-affecting factors, and cross-validation to control for selection bias were employed, residual confounders may persist. Specifically, the study design might be susceptible to biased indications, timing, and choice of CRRT modality. To overcome this limitation, we conducted an additional IPTW analysis, and the results were consistent with those of the multivariate analysis. Third, there were no timely data regarding the initiation of CRRT and ECMO. Determining the effects of early CRRT is a challenging issue in the management of CS patients with AKI. Fourth, there were no detailed data regarding the methods (independent CRRT access or introduction of a CRRT device into the ECMO circuit) of combining ECMO and CRRT. Our study dataset does not include information on heart transplantation events, which could be a significant clinical endpoint. Fifth, CKD was only classified based on medical records because of the nature of CS (emergent presentation and rapid progression), and estimating the true baseline creatinine value was impossible. Furthermore, we did not exclude patients with end-stage renal disease who were receiving hemodialysis due to a lack of clinical information.

## 5. Conclusions

In conclusion, patients with CS who underwent CRRT had higher 30-day all-cause mortality rates than those who did not undergo CRRT. Notably, in CS patients undergoing ECMO, CRRT was administered more frequently, suggesting the potential for CRRT to enhance early in-hospital clinical outcomes. However, further well-designed randomized controlled trials are warranted to assess the optimal CRRT strategies and their impact on clinical outcomes among CS patients receiving ECMO support.

## Figures and Tables

**Table 1:** Baseline characteristics.

	Without ECMO (N = 751)			With ECMO (N = 496)		
	Non-CRRT (N = 661)	CRRT (N = 90)	*p* Value	Non-CRRT (N = 301)	CRRT (N = 195)	*p* Value
Age (years)	67.4 ± 13.2	73.8 ± 9.9	<0.001	62.0 ± 14.4	61.5 ± 13.9	0.669
≥70	306 (46.3)	61 (67.8)	<0.001	96 (31.9)	57 (29.2)	0.598
Female	195 (29.5)	39 (43.3)	0.011	106 (35.2)	47 (24.1)	0.012
Cause of shock			0.085			0.979
ICMP	576 (87.1)	70 (77.8)		221 (73.4)	139 (71.3)	
Dilated cardiomyopathy	30 (4.5)	7 (7.8)		28 (9.3)	11 (5.6)	
Myocarditis	5 (0.8)	0 (0.0)		20 (6.6)	15 (7.7)	
Stress-induced cardiomyopathy	9 (1.4)	2 (2.2)		5 (1.7)	4 (2.1)	
Valvular heart disease	5 (0.8)	2 (2.2)		3 (1.0)	9 (4.6)	
Refractory VT/Vf	15 (2.3)	5 (5.6)		7 (2.3)	4 (2.1)	
PTE	9 (1.4)	1 (1.1)		8 (2.7)	6 (3.1)	
Other	12 (1.8)	3 (3.3)		9 (3.0)	7 (3.6)	
Medical history						
Hypertension	364 (55.1)	62 (68.9)	0.018	144 (47.8)	90 (46.2)	0.783
Diabetes mellitus	213 (32.2)	51 (56.7)	<0.001	109 (36.2)	70 (35.9)	1.000
Dyslipidemia	199 (30.1)	28 (31.1)	0.942	57 (18.9)	46 (23.6)	0.257
MI	80 (12.1)	18 (20.0)	0.055	38 (12.6)	24 (12.3)	1.000
Previous PCI	84 (12.7)	17 (18.9)	0.148	37 (12.3)	37 (19.0)	0.056
CABG	14 (2.1)	3 (3.3)	0.445	2 (0.7)	10 (5.1)	0.002
PAOD	24 (3.6)	11 (12.2)	0.001	9 (3.0)	8 (4.1)	0.680
Stroke	64 (9.7)	16 (17.8)	0.031	22 (7.3)	17 (8.7)	0.690
CKD	46 (7.0)	39 (43.3)	<0.001	8 (2.7)	30 (15.4)	0.001
Current smoker	211 (31.9)	10 (11.1)	<0.001	82 (27.2)	53 (27.2)	1.000
Treatment strategy			<0.001			0.049
PCI	497 (75.2)	44 (48.9)		182 (60.5)	103 (52.8)	
CABG	15 (2.3)	6 (6.7)		10 (3.3)	15 (7.7)	
No revascularization	149 (22.5)	40 (44.4)		109 (36.2)	77 (39.5)	
IABP	212 (32.1)	37 (41.1)	0.112	28 (9.3)	37 (19.0)	0.003
ECPR				139 (46.2)	101 (51.8)	0.258
Mechanical ventilation	228 (34.5)	72 (80.0)	<0.001	227 (75.4)	182 (93.3)	<0.001
Vasoactive inotropic score	52.4 ± 117.9	74.9 ± 97.3	0.046	102.1 ± 182.8	99.8 ± 113.7	0.867

The values are presented as the means ± SDs or numbers (percentages). CABG, coronary artery bypass graft; CKD, chronic kidney disease; CRRT, continuous renal replacement therapy; ECMO, extracorporeal membranous oxygenation; ECPR, extracorporeal cardiopulmonary resuscitation; IABP, intra-aortic balloon pump; ICMP, ischemic cardiomyopathy; MI, myocardial infarction; PAOD, peripheral arterial occlusive disease; PCI, percutaneous coronary intervention; PTE, pulmonary thromboembolism; VT/Vf, ventricular tachycardia/ventricular fibrillation.

**Table 2:** Clinical outcomes.

	Without ECMO (N = 751)			With ECMO (N = 496)		
	Non-CRRT (N = 661)	CRRT (N = 90)	*p* Value	Non-CRRT (N = 301)	CRRT (N = 195)	*p* Value
Length of stay (days)						
ICU stay (median [IQR])	3.0 [1.0, 7.0]	9.5 [4.0, 18.0]	<0.001	8.0 [2.0, 16.0]	11.0 [4.0, 20.0]	0.002
Hospital stay (median [IQR])	8.0 [4.0, 13.0]	18.0 [6.0, 35.0]	<0.001	14.0 [5.0, 29.0]	14.0 [5.2, 32.8]	0.227
Primary outcomes						
72 h all-cause mortality	50 (7.6)	10 (11.1)	0.339	59 (19.6)	24 (12.3)	0.045
30-day all-cause mortality	96 (14.5)	41 (45.6)	<0.001	109 (36.2)	125 (64.1)	<0.001
Secondary outcomes						
1-year all-cause mortality	122 (18.5)	55 (61.1)	<0.001	116 (38.5)	143 (73.3)	<0.001
1-year HF	27 (4.1)	3 (3.3)	1.00	3 (1.0)	3 (1.5)	0.684
1-year revascularization	10 (1.5)	1 (1.1)	1.00	3 (1.0)	3 (1.5)	0.684
1-year stroke	3 (0.5)	1 (1.1)	0.401	2 (0.7)	0 (0.0)	0.522

Values are presented as numbers (percentages) or medians (25th percentiles–75th percentiles). CRRT, continuous renal replacement therapy; CVA, cerebrovascular accident; ECMO, extracorporeal membranous oxygenation; IQR, interquartile range; HF, heart failure.

**Table 3:** Multivariate analysis of 72 h mortality in patients with cardiogenic shock stratified by the presence or absence of ECMO.

	Without ECMO		With ECMO	
	Adjusted HR (95% CI)	*p* Value	Adjusted HR (95% CI)	*p* Value
Age	1.01 (0.98, 1.03)	0.646	1.01 (0.98, 1.04)	0.703
Female	0.55 (0.27, 1.14)	0.11	1.03 (0.42, 2.52)	0.947
Hypertension	0.65 (0.33, 1.29)	0.218	1.28 (0.63, 2.63)	0.494
Diabetes	1.64 (0.83, 3.22)	0.154	0.73 (0.36, 1.49)	0.384
Previous MI	0.17 (0.02, 1.27)	0.085	0.47 (0.14, 1.62)	0.233
Previous PAOD	0.45 (0.06, 3.51)	0.45	0.36 (0.05, 2.83)	0.331
Previous stroke	2.18 (0.95, 5.02)	0.067	0.44 (0.1, 1.99)	0.287
Ischemic cardiomyopathy	1.31 (0.57, 3.01)	0.52	2.47 (0.88, 6.91)	0.085
Lactate	1.16 (1.09, 1.23)	<0.001	1.07 (1, 1.15)	0.043
eGFR (mL/min/1.73 m^2^)				
≥70	ref			
45–69	0.71 (0.28, 1.79)	0.471	0.95 (0.32, 2.78)	0.922
30–44	0.67 (0.24, 1.86)	0.439	1.16 (0.37, 3.59)	0.798
<30	1.14 (0.42, 3.08)	0.802	1.83 (0.52, 6.38)	0.344
Vasoactive inotropic score	1 (1, 1)	<0.001	1 (1.001, 1.002)	0.005
IABP	1.27 (0.65, 2.47)	0.488		
ECPR			1.83 (0.87, 3.88)	0.113
Mechanical ventilation	2.02 (0.95, 4.28)	0.066	7.82 (1.02, 59.95)	0.048
CRRT	0.57 (0.24, 1.35)	0.203	0.44 (0.21, 0.91)	0.027

CI, confidence interval; CRRT, continuous renal replacement therapy; ECMO, extracorporeal membranous oxygenation; ECPR, extracorporeal cardiopulmonary resuscitation; eGFR, estimated glomerular filtration rate; HR, hazard ratio; IABP, intra-aortic balloon pump; MI, myocardial infarction; PAOD, peripheral arterial occlusive disease.

**Table 4:** Multivariate analysis for 1-month mortality in patients with cardiogenic shock according to ECMO status.

	Without ECMO		With ECMO	
	Adjusted HR (95% CI)	*p* Value	Adjusted HR (95% CI)	*p* Value
Age	1.03 (1.01, 1.05)	0.011	1 (0.98, 1.02)	0.986
Female	0.67 (0.42, 1.08)	0.1	0.83 (0.47, 1.46)	0.511
Hypertension	0.76 (0.47, 1.22)	0.252	1.32 (0.84, 2.07)	0.233
Diabetes	1.76 (1.11, 2.78)	0.016	0.9 (0.57, 1.41)	0.639
Previous MI	0.6 (0.3, 1.18)	0.138	0.86 (0.46, 1.59)	0.625
Previous PAOD	1.03 (0.43, 2.46)	0.942	1.41 (0.55, 3.58)	0.471
Previous CVA	1.08 (0.6, 1.95)	0.796	1.29 (0.62, 2.66)	0.5
Ischemic cardiomyopathy	1.54 (0.86, 2.78)	0.149	1.34 (0.77, 2.35)	0.3
Lactate	1.13 (1.08, 1.17)	<0.001	1.06 (1.01, 1.11)	0.016
eGFR (mL/min/1.73 m^2^)				
≥70	ref			
45–69	0.96 (0.49, 1.88)	0.915	1.05 (0.54, 2.03)	0.895
30–44	1.04 (0.52, 2.09)	0.902	0.97 (0.48, 1.98)	0.94
<30	1.01 (0.49, 2.08)	0.972	0.79 (0.35, 1.81)	0.579
Vasoactive inotropic score	1 (1, 1)	<0.001	1 (1.001, 1.002)	<0.001
IABP	1.28 (0.83, 1.98)	0.271		
ECPR			2.4 (1.53, 3.77)	< 0.001
Mechanical ventilation	1.73 (1.05, 2.84)	0.03	1.14 (0.72, 1.8)	0.578
CRRT	1.45 (0.87, 2.41)	0.151	2.63 (1.66, 4.16)	<0.001

CI, confidence interval; CRRT, continuous renal replacement therapy; ECMO, extracorporeal membranous oxygenation; ECPR, extracorporeal cardiopulmonary resuscitation; eGFR, estimated glomerular filtration rate; HR, hazard ratio; IABP, intra-aortic balloon pump; MI, myocardial infarction; PAOD, peripheral arterial occlusive disease.

**Table 5:** Comparison of impact of CRRT on mortality according to ECMO status by multivariate Cox regression and IPTW.

	Multivariate Cox Regression		IPTW	
	Adjusted HR (95% CI)	*p* Value	Adjusted HR (95% CI)	*p* Value
Without ECMO				
72 h mortality	0.57 (0.24, 1.35)	0.203	0.65 (0.23, 1.83)	0.415
1-month mortality	1.45 (0.87, 2.41)	0.151	1.80 (0.98, 3.32)	0.059
With ECMO				
72 h mortality	0.44 (0.21, 0.91)	0.027	0.40 (0.19, 0.86)	0.019
1-month mortality	2.63 (1.66, 4.16)	<0.001	1.77 (1.17, 2.67)	0.006

CI, confidence interval; CRRT, continuous renal replacement therapy; ECMO, extracorporeal membranous oxygenation; HR, hazard ratio; IPTW, inverse probability of treatment weighting.

**Table 6:** Independent predictors for CRRT.

	Univariate Analysis		Multivariate Analysis	
	OR (95% CI)	*p* Value	OR (95% CI)	*p* Value
Age	1.00 (0.99, 1.01)	0.711		
Female	1.05 (0.79, 1.4)	0.721		
Hypertension	1.02 (0.78, 1.33)	0.876		
Diabetes	1.47 (1.12, 1.92)	0.006		
Previous MI	1.24 (0.85, 1.81)	0.274		
Previous PAOD	2.01 (1.13, 3.59)	0.018		
Previous CVA	1.33 (0.87, 2.04)	0.184		
Ischemic cardiomyopathy	0.57 (0.42, 0.78)	<0.001	0.69 (0.45, 1.06)	0.087
Lactate	1.11 (1.07, 1.15)	<0.001	1.06 (1.01, 1.1)	0.008
eGFR (mL/min/1.73 m^2^)				
≥70	ref			
45–69	1.44 (0.89, 2.32)	0.136	1.46 (0.79, 2.73)	0.23
30–44	2.61 (1.63, 4.19)	<0.001	2.3 (1.24, 4.27)	0.008
<30	8.91 (5.58, 14.22)	<0.001	9.39 (5.02, 17.57)	<0.001
Vasoactive inotropic score	1.00 (1, 1)	0.015		
IABP	1.06 (0.78, 1.43)	0.728	1.46 (0.91, 2.32)	0.113
ECMO	4.76 (3.58, 6.32)	<0.001	3.59 (2.4, 5.38)	<0.001
ECPR	3.25 (2.4, 4.4)	<0.001		
Mechanical ventilation	1.04 (0.72, 1.49)	0.853	4.16 (2.51, 6.91)	<0.001

CI, confidence interval; CRRT, continuous renal replacement therapy; ECMO, extracorporeal membranous oxygenation; ECPR, extracorporeal cardiopulmonary resuscitation; eGFR, estimated glomerular filtration rate; IABP, intra-aortic balloon pump; OR, odds ratio; MI, myocardial infarction; PAOD, peripheral arterial occlusive disease.

## Data Availability

The data included in this study are available from the corresponding authors upon reasonable request.

## Supplementary Materials

The following supporting information can be downloaded at: https://www.mdpi.com/article/10.3390/jcm14051498/s1, Table S1. Laboratory data according to ECMO status; Table S2. Clinical outcomes according to CRRT status; Table S3. Baseline characteristics after adjustment with inverse-probability-of-treatment weighting (IPTW); Figure S1. Time-to-event curves for the primary outcomes in the overall cohort of patients.

## References

[1] van Diepen S., Katz J.N., Albert N.M., Henry T.D., Jacobs A.K., Kapur N.K., Kilic A., Menon V., Ohman E.M., Sweitzer N.K. (2017). Contemporary Management of Cardiogenic Shock: A Scientific Statement From the American Heart Association. Circulation.

[2] Ghionzoli N., Sciaccaluga C., Mandoli G.E., Vergaro G., Gentile F., D’Ascenzi F., Mondillo S., Emdin M., Valente S., Cameli M. (2021). Cardiogenic shock and acute kidney injury: The rule rather than the exception. Heart Fail. Rev..

[3] Libório A.B., Leite T.T., Neves F.M.d.O., Teles F., Bezerra C.T.d.M. (2015). AKI Complications in Critically Ill Patients: Association with Mortality Rates and RRT. Clin. J. Am. Soc. Nephrol..

[4] Rangaswami J., Bhalla V., Blair J.E.A., Chang T.I., Costa S., Lentine K.L., Lerma E.V., Mezue K., Molitch M., Mullens W. (2019). Cardiorenal Syndrome: Classification, Pathophysiology, Diagnosis, and Treatment Strategies: A Scientific Statement From the American Heart Association. Circulation.

[5] Adegbala O., Inampudi C., Adejumo A., Otuonye G., Akintoye E., Elsayed R., Williams K., Alvarez P., Afonso L., Briasoulis A. (2019). Characteristics and Outcomes of Patients With Cardiogenic Shock Utilizing Hemodialysis for Acute Kidney Injury. Am. J. Cardiol..

[6] Selewski D.T., Wille K.M. (2021). Continuous renal replacement therapy in patients treated with extracorporeal membrane oxygenation. Semin. Dial..

[7] Fleming G.M., Askenazi D.J., Bridges B.C., Cooper D.S., Paden M.L., Selewski D.T., Zappitelli M. (2012). A multicenter international survey of renal supportive therapy during ECMO: The Kidney Intervention During Extracorporeal Membrane Oxygenation (KIDMO) group. ASAIO J..

[8] Askenazi D.J., Selewski D.T., Paden M.L., Cooper D.S., Bridges B.C., Zappitelli M., Fleming G.M. (2012). Renal replacement therapy in critically ill patients receiving extracorporeal membrane oxygenation. Clin. J. Am. Soc. Nephrol..

[9] Schmidt M., Bailey M., Kelly J., Hodgson C., Cooper D.J., Scheinkestel C., Pellegrino V., Bellomo R., Pilcher D. (2014). Impact of fluid balance on outcome of adult patients treated with extracorporeal membrane oxygenation. Intensive Care Med..

[10] Yang J.H., Choi K.H., Ko Y.G., Ahn C.M., Yu C.W., Chun W.J., Jang W.J., Kim H.J., Kim B.S., Bae J.W. (2021). Clinical Characteristics and Predictors of In-Hospital Mortality in Patients With Cardiogenic Shock: Results From the RESCUE Registry. Circ. Heart Fail..

[11] Jun M., Bellomo R., Cass A., Gallagher M., Lo S., Lee J. (2014). Timing of renal replacement therapy and patient outcomes in the randomized evaluation of normal versus augmented level of replacement therapy study. Crit. Care Med..

[12] Mandelbaum T., Scott D.J., Lee J., Mark R.G., Malhotra A., Waikar S.S., Howell M.D., Talmor D. (2011). Outcome of critically ill patients with acute kidney injury using the Acute Kidney Injury Network criteria. Crit. Care Med..

[13] McDonagh T.A., Metra M., Adamo M., Gardner R.S., Baumbach A., Böhm M., Burri H., Butler J., Čelutkienė J., Chioncel O. (2021). 2021 ESC Guidelines for the diagnosis and treatment of acute and chronic heart failure. Eur. Heart J..

[14] (2017). ELSO Guidelines for Cardiopulmonary Extracorporeal Life Support.

[15] Fuernau G., Poenisch C., Eitel I., Denks D., de Waha S., Pöss J., Heine G.H., Desch S., Schuler G., Adams V. (2015). Prognostic impact of established and novel renal function biomarkers in myocardial infarction with cardiogenic shock: A biomarker substudy of the IABP-SHOCK II-trial. Int. J. Cardiol..

[16] Tigano S., Caruso A., Liotta C., LaVia L., Vargas M., Romagnoli S., Landoni G., Sanfilippo F. (2024). Exposure to severe hyperoxemia worsens survival and neurological outcome in patients supported by veno-arterial extracorporeal membrane oxygenation: A meta-analysis. Resuscitation.

[17] Shaefi S., O’Gara B., Kociol R.D., Joynt K., Mueller A., Nizamuddin J., Mahmood E., Talmor D., Shahul S. (2015). Effect of cardiogenic shock hospital volume on mortality in patients with cardiogenic shock. J. Am. Heart Assoc..

[18] Veyret S., Girard L., Puech B., Dangers L., Jabot J., Neuschwander A., Braunberger E., Allyn J., Allou N., Vidal C. (2024). The IMPACT Score: A New Score to Predict the Risk of Early Mortality in Cardiogenic Shock Patients Treated With Venoarterial Extracorporeal Membrane Oxygenation. J. Cardiothorac. Vasc. Anesth..

[19] Smith M., Vukomanovic A., Brodie D., Thiagarajan R., Rycus P., Buscher H. (2017). Duration of veno-arterial extracorporeal life support (VA ECMO) and outcome: An analysis of the Extracorporeal Life Support Organization (ELSO) registry. Crit. Care.

[20] Chen H., Yu R.G., Yin N.N., Zhou J.X. (2014). Combination of extracorporeal membrane oxygenation and continuous renal replacement therapy in critically ill patients: A systematic review. Crit. Care.

[21] Lauridsen M.D., Gammelager H., Schmidt M., Rasmussen T.B., Shaw R.E., Bøtker H.E., Sørensen H.T., Christiansen C.F. (2015). Acute kidney injury treated with renal replacement therapy and 5-year mortality after myocardial infarction-related cardiogenic shock: A nationwide population-based cohort study. Crit. Care.

[22] Chen S.W., Lu Y.A., Lee C.C., Chou A.H., Wu V.C., Chang S.W., Fan P.C., Tian Y.C., Tsai F.C., Chang C.H. (2019). Long-term outcomes after extracorporeal membrane oxygenation in patients with dialysis-requiring acute kidney injury: A cohort study. PLoS ONE.

[23] Núñez J., Miñana G., Santas E., Bertomeu-González V. (2015). Cardiorenal Syndrome in Acute Heart Failure: Revisiting Paradigms. Rev. Esp. Cardiol. (Engl. Ed.).

[24] Berl T., Henrich W. (2006). Kidney-heart interactions: Epidemiology, pathogenesis, and treatment. Clin. J. Am. Soc. Nephrol..

[25] Caetano F., Barra S., Faustino A., Botelho A., Mota P., Costa M., Leitão Marques A. (2014). Cardiorenal syndrome in acute heart failure: A vicious cycle?. Rev. Port. Cardiol..

