# Supplementary material for: Continuous Renal Replacement Therapy and Extracorporeal Membrane Oxygenation in Patients with Cardiogenic Shock: Results from the Rescue Registry
Source: J Clin Med. 2025 Feb 24;14(5):1498. doi: 10.3390/jcm14051498 (PMC11900090; doi:10.3390/jcm14051498)
Supplement: Supplementary file 1 [file jcm-14-01498-s001.zip › jcm-3468554-supplementary.pdf]

Supplementary Table S1. Laboratory data

	Without ECMO (N=751)			With ECMO (N=496)		
	**Non-CRRT (N=661)**	**CRRT (N=90)**	**P Value**	**Non-CRRT (N=301)**	**CRRT (N=195)**	**P Value**
**Hemoglobin (g/dL)**	13.0 ± 2.4	11.3 ± 2.4	<0.001	12.5 ± 2.7	12.3 ± 2.9	0.292
**Platelet count (1000//µL)**	222.8 ± 77.5	199.4 ± 80.8	0.011	209.7 ± 86.6	178.0 ± 77.8	<0.001
**Total bilirubin, mg/dL**	0.9 ± 0.8	1.2 ± 1.2	0.01	1.0 ± 1.2	1.5 ± 3.8	0.058
**Aspartate aminotransferase (IU/dL)**	154.0 ± 448.1	666.0 ± 2426.6	0.051	247.1 ± 860.8	432.6 ± 1267.3	0.079
**Alanine aminotransferase (IU/dL)**	96.4 ± 321.8	318.6 ± 957.6	0.033	141.4 ± 432.2	230.3 ± 526.7	0.054
**Sodium (mmol/L)**	137.9 ± 4.8	135.1 ± 5.2	<0.001	137.0 ± 5.8	136.6 ± 6.8	0.561
**Creatinine**	1.3 ± 1.1	3.0 ± 2.3	<0.001	1.3 ± 0.7	2.0 ± 1.8	<0.001
**eGFR (mL/min/1.73 m2)**	59.9 ± 35.2	29.2 ± 20.1	<0.001	57.6 ± 31.5	46.5 ± 50.9	0.008
**≥ 70**	171 (26.5)	4 (4.4)		68 (23.0)	24 (12.7)	
**45-69**	246 (38.1)	12 (13.3)		110 (37.2)	48 (25.4)	
**30-44**	144 (22.3)	21 (23.3)		88 (29.7)	50 (26.5)	
**< 30**	85 (13.2)	53 (58.9)		30 (10.1)	67 (35.4)	

**Lactate (mmol/L)**	5.5 ± 4.1	7.6 ± 5.2	0.003	7.1 ± 4.3	8.7 ± 4.7	0.002
**Lactate > 5**	154 (40.3)	42 (61.8)	0.002	125 (61.6)	99 (71.7)	0.068
**LVEF shock date**	40.0 ± 13.7	35.3 ± 15.0	0.059	26.0 ± 14.9	27.3 ± 15.2	0.551
**Peak CK-MB (μg/dL)**	160.7 ± 321.7	131.1 ± 171.7	0.181	189.9 ± 277.1	266.3 ± 614.6	0.108
**Peak Troponin I (ng/mL)**	46.8 ± 121.8	64.5 ± 188.1	0.392	61.7 ± 122.9	72.1 ± 167.8	0.470
**NT-proBNP (pg/mL)**	6028.8 ± 9543.7	22112.2 ± 17017.6	<0.001	8916.3 ± 13337.3	10810.2 ± 12124.5	0.212

The values are presented as the means ± SDs or numbers (percentages). LVEF, left ventricular ejection fraction; NT-proBNP, N-terminal pro-B-type natriuretic peptide.

Supplementary Table S2. Clinical outcomes according to CRRT status

	Non-CRRT (N=962)	CRRT (N=285)	P Value
**Length of stay (days)**			
**ICU stay (median [IQR])**	4.0 [2.0, 10.0]	11.0 [4.0, 20.0]	<0.001
**Hospital stay (median [IQR])**	9.0 [4.0, 17.0]	16.0 [5.8, 33.2]	<0.001
**Primary outcomes**			
**72-hour all-cause mortality**	109 (11.3)	31 (10.9)	0.915
**30-day all-cause mortality**	205 (21.3)	166 (58.2)	<0.001
**Secondary outcomes**			
**1-year all-cause mortality**	238 (24.7)	198 (69.5)	<0.001
**1-year HF**	30 (3.1)	6 (2.1)	0.486
**1-year revascularization**	13 (1.4)	4 (1.4)	1.00
**1-year stroke**	5 (0.5)	1 (0.4)	1.00

Values are presented as number (percentage), or median [25th percentiles-75th percentile]. CRRT, continuous renal replacement therapy; CVA, cerebrovascular accident; ECMO, extracorporeal membranous oxygenation; IQR, interquartile range; HF, heart failure.

Supplementary Table S3. Baseline characteristics after adjustment with inverse-probability-of-treatment weighting (IPTW)

	Without ECMO (N=799)	With ECMO (N=776)	*p* value	SMD
**Age**	65.0 ± 15.6	65.2 ± 14.3	0.92	0.010
**Female**	277 (34.6)	265 (34.1)	0.91	0.011
**Hypertension**	408 (51.0)	397 (51.1)	0.98	0.003
**Diabetes**	290 (36.3)	289 (37.2)	0.84	0.017
**Previous MI**	98 (12.2)	88 (11.3)	0.73	0.028
**Previous PAOD**	38 (4.8)	48 (6.2)	0.53	0.061
**Previous CVA**	86 (10.7)	89 (11.4)	0.81	0.023
**Ischemic cardiomyopathy**	583 (72.9)	581 (74.9)	0.61	0.046
**Lactate**	7.1 ± 5.2	7.2 ± 4.2	0.86	0.018
**eGFR (mL/min/1.73 m2)**			0.98	0.037
**≥ 70**	147 (18.4)	133 (17.2)		
**45-69**	252 (31.5)	248 (31.9)		
**30-44**	218 (27.3)	221 (28.4)		
**< 30**	182 (22.8)	174 (22.5)		

**Vasoactive inotropic score**	94.9 ± 202.0	85.4 ± 147.1	0.61	0.054
**Mechanical ventilation**	512 (64.0)	507 (65.4)	0.76	0.028
**CRRT**	198 (24.8)	203 (26.2)	0.73	0.031

The values are presented as the means ± SDs or numbers (percentages). CRRT, continuous renal replacement therapy; CVA, cerebrovascular accident; ECMO, extracorporeal membranous oxygenation; eGFR, estimated glomerular filtration rate; MI, myocardial infarction; PAOD, peripheral arterial occlusive disease; SMD, standardized mean difference.

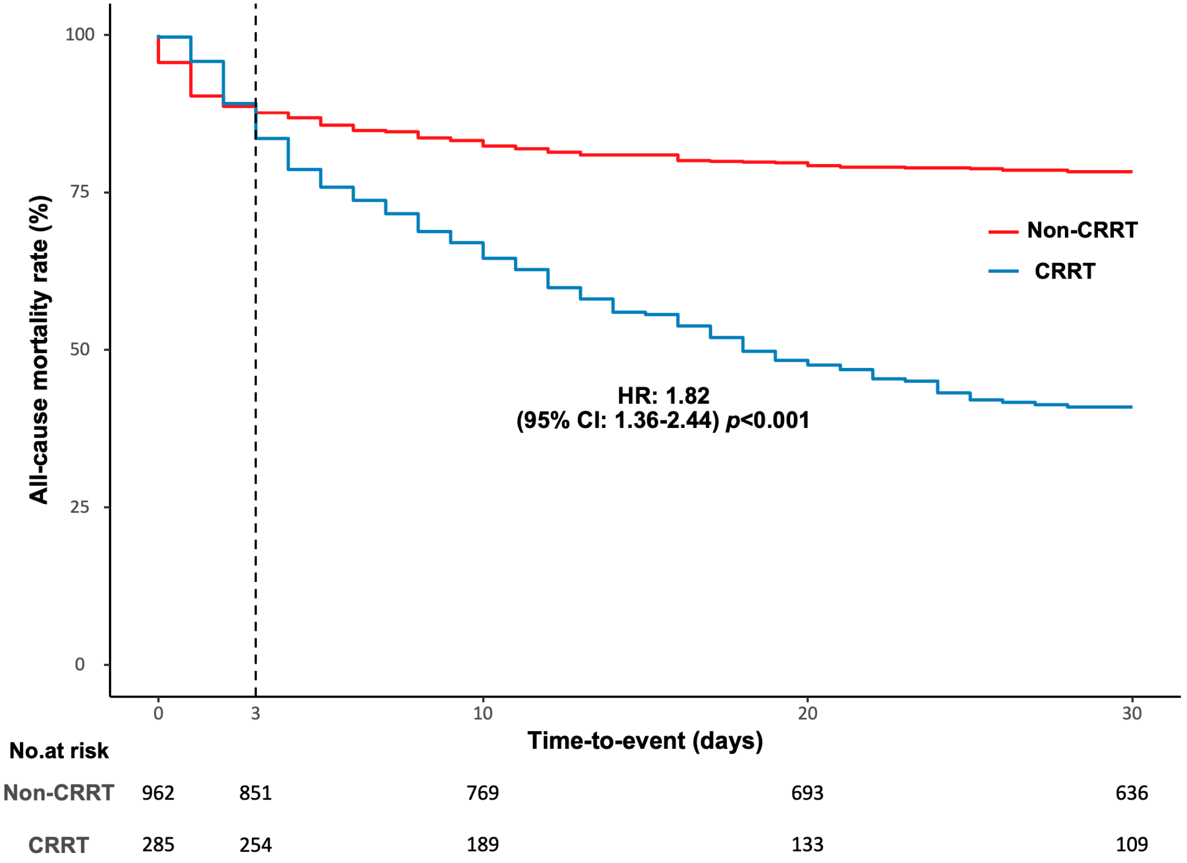

Supplementary Figure S1. Time-to-event curves for the primary outcomes in the overall cohort of patients

CI, confidence interval; CRRT, continuous renal replacement therapy; ECMO, extracorporeal membrane oxygenation; HR, hazard ratio
